# Feto-Maternal Outcomes in Pregnancy With Factor VII Deficiency in a Tertiary Care Institution

**DOI:** 10.7759/cureus.64079

**Published:** 2024-07-08

**Authors:** Mohini Sachdeva, Amanjot Kaur, Neelam Aggarwal, Jasmina Ahluwalia, Simran Vohra

**Affiliations:** 1 Obstetrics and Gynecology, Postgraduate Institute of Medical Education and Research, Chandigarh, IND; 2 Hematology, Postgraduate Institute of Medical Education and Research, Chandigarh, IND

**Keywords:** factor 7, anesthesia in factor 7 deficiency, delivery in patient with factor 7 deficiency, pregnancy in patient with factor 7 deficiency, factor 7 deficiency

## Abstract

This paper aims to study antepartum and postpartum bleeding manifestations of patients with factor VII (FVII) deficiency, their management, and feto-maternal outcomes, to establish danger signs and management protocols.

We describe a case series of nine pregnancies in four patients with FVII deficiency diagnosed at a tertiary care referral center in India between 2012 and 2023. Out of nine pregnancies, six had cesarean deliveries, two had vaginal deliveries, and one had dilatation and curettage for unwanted pregnancies. One out of nine pregnancies (11.11%) with an unknown FVII deficiency had antepartum hemorrhage (abruption) necessitating multiple transfusions, ICU stay, and neonatal loss. Three patients with no prior history of obstetric hemorrhage were diagnosed with severe deficiencies and received prophylactic recombinant FVII preoperatively, averting the potential loss of lives. In patients with no history of bleeding, no hemorrhage was reported with or without prophylaxis while 33.33% of hemorrhage was reported in patients with a history of bleeding.

Factors like the history of bleeding, FVII levels, mode of delivery, and other risk factors for hemorrhage should all be considered to predict the risk of bleeding in delivery. Cesarean is a surgical procedure, and prophylactic use of recombinant FVII concentrate (rFVIIa) should be considered.

## Introduction

Factor VII (FVII), also known as Proconvertin, is a vitamin K-dependent protein. Synthesized exclusively by the liver, its plasma levels range around 0.35 to 0.60 mg/L (normal coagulant activity varies between 70% and 140%). Its half-life is extremely short (4-6 hours).

The prevalence of FVII deficiency is one in 50,0000. It is inherited in an autosomal-recessive fashion and results from a mutation in the F7 gene. It is higher in areas with high consanguinity rates. Heterozygotes have approximately half-normal levels of coagulation factors and, thus, are usually asymptomatic [1]. FVII deficiency is associated with prolongation of prothrombin time (PT) with normal activated partial thromboplastin (APTT) time in the coagulation profile and is confirmed with mixing studies [2].

FVII deficiency in reproductive-age women can manifest as menorrhagia, postabortal bleeding, and antepartum or postpartum hemorrhage (PPH). The International Society on Thrombosis and Hemostasis classifies FVII deficiency as follows: mild (20%-50%, usually asymptomatic), moderate (10%-20%, at risk for mild spontaneous or triggered bleeding), and severe (FVII <10%, greatest risk for major spontaneous bleeding). The risk of bleeding correlates poorly with the coagulation factor activity level for FVII as sometimes a patient with severe FVII deficiency may not have significant hemorrhage while a patient not severely deficient in FVII may hemorrhage profusely [3]. Still severe FVII deficiencies are associated with maximum risk of bleeding [4]. Thus, the risk of bleeding and PPH in these patients makes the discussion about FVII deficiency and its replacement and prophylaxis relevant. 

The type of prophylaxis used in FVII-deficient patients is fresh frozen plasma (FFP) and recombinant FVII concentrate (rFVIIa). FFPs are cost-effective and easily available but carry the risk of infections and volume overload (dose 15-20 mL/kg). rfVIIa is not widely available and is expensive. There are less than 90 cases reported worldwide in literature, and the dearth of literature further fuels ambiguity on whether all severe FVII deficiency cases have hemorrhage and need empiric prophylaxis before delivery. Thus, we decided to analyze our experience of pregnancy with FVII deficiency, their fetomaternal outcome, the correlation of hemorrhage with FVII levels, and the impact of prophylaxis with rfVIIa and FFPs.

## Case presentation

We report four cases and nine pregnancies of FVII deficiency booked over the last 11 years in our institute (2012-2023). A bleeding history was considered positive if previous menorrhagia, epistaxis, post-traumatic or postsurgical bleeding was reported. PPH was defined as >500 mL blood loss with vaginal delivery or >1,000 mL blood loss with cesarean section. 

Case 1

A 28-year-old third gravida (G3P1L1A1) with hepatitis C-positive status was admitted at 37 weeks of gestation in early labor. She was diagnosed with FVII deficiency in her first pregnancy when she was evaluated for a deranged coagulogram. Her antenatal period was uneventful. She did not have any previous history of gum bleeding, epistaxis, or excessive bleeding following injury. She was diagnosed with hepatitis C in her previous pregnancy (HCV RNA 7180 copies per mL), with no evidence of chronic liver disease, and was under hepatology follow-up. Her first pregnancy six years ago ended in a term vaginal delivery with no obstetric hemorrhage or blood transfusion. Subsequently, she had a dilatation and curettage for an unwanted pregnancy with no history of postabortal hemorrhage. Her baseline FVII level was 7.9%, and her current level of FVII was 2.5%. She received 3 units of FFPs transfusion and Tranexamic acid as prophylaxis. She delivered a 3.1 kg baby girl vaginally with a first-degree perineal tear, with a blood loss of 200 mL. Family screening for FVII deficiency, coagulogram, HCV RNA, and FVII of the child was advised. The baby and mother were discharged on day 3 of delivery.

Case 2

A 24-year-old primigravida at 31 weeks 4 days gestation was referred with severe pre-eclampsia, placental abruption, and disseminated intravascular coagulation. The patient had a history of multiple episodes of bleeding per vaginum (mild) in the second trimester. She also had a history of epistaxis and gum bleeding on and off since childhood but was never evaluated for the same. The patient received 5 FFPs and 1 unit packed red blood cell (PRBC) at the referring hospital. On admission, her investigations revealed hemoglobin of 7.4 g/dL, platelet count of 70,000/microL, PT of 35 seconds, activated partial thromboplastin (APTT) of 29 seconds, and international normalized ratio (INR) of 2.6. An emergency cesarean section was done for massive antepartum hemorrhage under general anesthesia. Intraoperatively, liquor was blood-stained, and 1 kg retroplacental clots were evacuated. The patient was shifted in an intubated state to the ICU and received intravenous antibiotics, magnesium sulfate, multiple FFPs, and PRBCs during the ICU stay. She was extubated on the fifth postoperative and was discharged on day 8. Baby expired on day 8 of life. The patient was subsequently evaluated by hematology for bleeding disorders due to persistent coagulation abnormalities unexplained by abruption and diagnosed with FVII deficiency (level 0.7%).

Case 3

A 29-year-old second gravida (G2P1L1) at 37 weeks with a previous cesarean section and newly diagnosed Factor VII deficiency (incidentally diagnosed in this pregnancy after an abnormal coagulation profile). Her antenatal period was uneventful. She had a history of gum bleeding on and off. Previous pregnancy concluded with a term cesarean section given fetal distress with meconium-stained liquor with no history of hemorrhage or blood transfusion. FVII level in the current pregnancy was 0.8%. Labor was induced in this pregnancy with a mechanical method, but the patient underwent an emergency cesarean given fetal distress under general anesthesia. She received recombinant FVII preoperatively. The blood loss was 500 mL.

Case 4

A 25-year-old third gravida (G3P2L2) at 38 weeks six-day gestation with two previous cesarean sections was referred to our institution with a deranged coagulation profile (prolonged PT). The patient did not have any antenatal complications or history of bleeding manifestations in the past or during the previous two cesarean sections. There was no family history suggestive of a bleeding disorder. A repeat coagulogram performed at our institution reported PT 33.9 and INR 2.31. A mixing study assay confirmed FVII deficiency (5.5%). The patient underwent elective cesarean section with tubal ligation under general anesthesia at 39 weeks due to the previous two cesarean sections. Prophylactic rFVIIa was administered preoperatively. Blood loss was 250 mL.

We had four women and a total of nine pregnancies, six cesarean deliveries, two vaginal deliveries, and one dilatation and curettage for unwanted pregnancy. Two patients (cases 2 and 3) had a history of bleeding manifestations before pregnancy and two (cases 1 and 4) had no bleeding manifestations. The mean FVII level was 0.75% in the bleeding group as compared to 4% in the nonbleeding group (range 0.7%-5.5%). There were three pregnancies in the bleeding diathesis group out of which antepartum hemorrhage was seen in one pregnancy (33.33%). In the nonbleeding diathesis group, six pregnancies (four without any prophylaxis while undiagnosed) and no hemorrhage were observed.

One out of nine pregnancies (11.11%) with an unknown FVII deficiency had antepartum hemorrhage (abruption), necessitating multiple transfusions of blood and blood products, perioperatively, ICU stay, and neonatal loss due to prematurity. No hemorrhage-related complications were reported in the other eight pregnancies. rfVIIa was the preferred prophylaxis (66% of cases and all were cesarean deliveries), while FFPs and tranexamic acid were the preferred prophylaxis in vaginal deliveries (used in 33% of cases, all vaginal deliveries). Prophylaxis was 100% effective in averting a major obstetrical hemorrhage. Interestingly, all women who received prophylaxis were diagnosed with severe FVII deficiency and had a previous obstetric history with no obstetric hemorrhage without any prophylaxis (Table 1).

**Table 1:** Details of pregnancies with factor 7 deficiency. rfVIIa, recombinant factor VII concentrate; FFP, fresh frozen plasma

Patient	Pregnancies	Bleeding diathesis	Factor VII	Mode of delivery	Hemorrhage and prophylaxis	Baby
Case 1	P1	No	Diagnosed and levels unavailable	Vaginal	No hemorrhage	Alive
	P2		Levels unavailable	Dilatation and currettage	No hemorrhage	
	P3		2.5%	Vaginal	No hemorrhage; prophylaxis with FFPs and tranexamic acid	alive
Case 2	P1	Yes	0.7% (diagnosed post-delivery)	Cesarean at 31 weeks, given severe pre-eclampsia with abruption	Antepartum hemorrhage and postpartum hemorrhage; no rfVIIa received, multiple blood and blood products intra-op and post-op	Expired on day 8
Case 3	P1	Yes	Undiagnosed	Cesarean	No hemorrhage	Alive
	P2		0.8%	Cesarean	No hemorrhage; prophylaxis with rfVIIa	Alive
Case 4	P1	No	Undiagnosed	Cesarean	No hemorrhage	Alive
	P2		Undiagnosed	Cesarean	No hemorrhage	Alive
	P3		5.5%	Cesarean with tubal ligation	No hemorrhage; received rfVIIa and tranexamic acid prophylaxis	Alive

## Discussion

**Figure 1:**
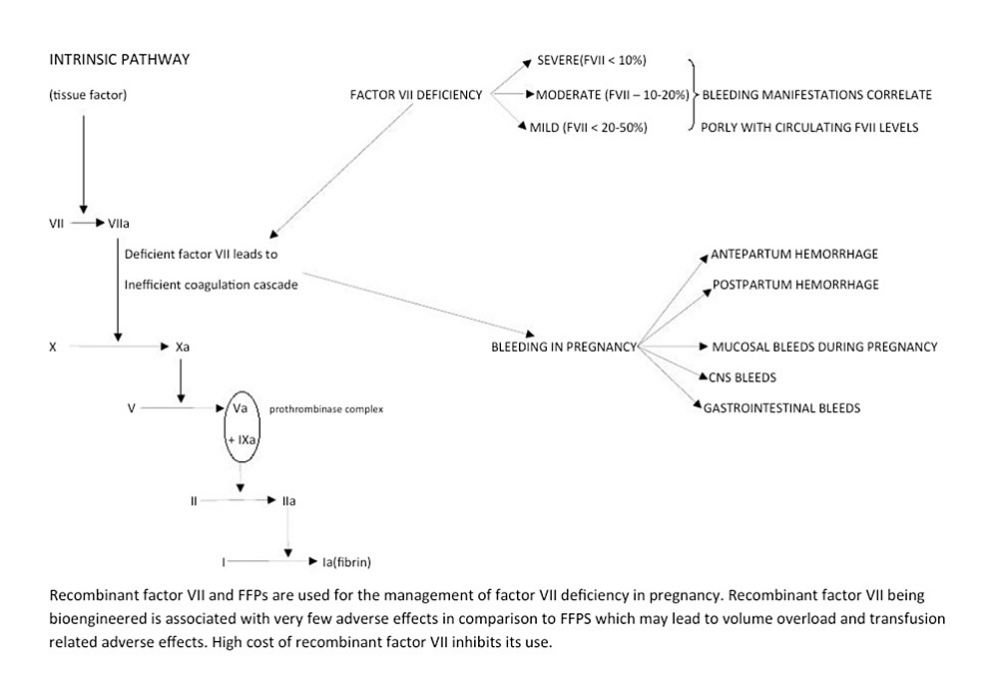
Flowchart showing the role of factor VII in coagulation cascade, its deficiency spectrum, and presentation and replacement therapy. Image credit: Amanjot Kaur. FFP, fresh frozen plasma; rfVIIa, recombinant factor VII concentrate

Replacement therapy for FVII deficiency requires consideration of several factors: FVII levels, whether it is being used for prophylactic purposes, or therapeutic indications such as spontaneous intracranial bleeds and gastrointestinal bleeds. Cost is another important factor to keep in mind when deciding on replacement therapy. Various options available for replacement therapy include rFVIIa, FFP (dose 15-30 mL/kg), and plasma-derived FVII concentrates (dose 15-40 IU/kg). rFVIIa is most commonly used. FFPs and plasma-derived FVII concentrates can be used in places where rFVIIa is not available or is unaffordable but needs to be transfused in large volumes to get the desired results, leading to volume overload. Other complications may include transfusion-related acute lung injury (TRALI) and transmission of viral infections (Figure 1). The dose of rFVIIa varies according to indication and may vary from 20 to 120 microg/kg. All patients diagnosed with severe FVII deficiency (<10%) received prophylaxis in our institute as policy irrespective of bleeding history and mode of delivery. We noticed a trend that rfVIIa was preferred for prophylaxis in cesarean deliveries and FFPs preferred for vaginal deliveries. Thus, the mode of delivery (cesarean being a major surgical procedure) inadvertently influenced the type of prophylaxis, which could be attributed to the high cost and restricted availability of rfVIIa in a poor resource country like ours.

In the nonbleeding diathesis group (mean FVII level 4%), no obstetric hemorrhage was observed in either subset: those who received prophylaxis (2/6) and those without prophylaxis (4/6). Cases 3 and 4 underwent cesareans in previous pregnancies without any prophylaxis (while they were undiagnosed with FVII deficiency) and had no obstetric hemorrhage. Kreuziger et al. conducted a meta-analysis in 2013 and observed that no postpartum hemorrhages (PPHs) were reported in women who lacked a bleeding history [5]. On analyzing published data (Table 2) after the publication of the meta-analysis by Kreuziger et al., 12 case reports (19 pregnancies discussing pregnancy with FVII deficiency) can be identified. These data are tabulated in Table 2. The majority of these patients had a bleeding phenotype and had delivered vaginally(58%). Of the 10 vaginal deliveries, PPH was observed in 3 pregnancies. rFVIIa was not administered in any of these pregnancies, however, FFP was administered in one. Out of seven pregnancies that resulted in a cesarean section, one had an antepartum hemorrhage (APH) and one had a post-partum hemorrhage. While the details of replacement therapy for the patient having APH are not available, the one having PPH had received prophylaxis. Thus, the benefit of empirical prophylaxis in the nonbleeding subset could not be established as FVII levels failed to predict bleeding. 

**Table 2:** Previously published literature with factor 7 deficiency in pregnancy. rfVIIa, recombinant factor VII concentrate; FFP, fresh frozen plasma; PPH, postpartum hemorrhage

Author	Year	No. of women (No. of pregnancies)	Factor VII levels	Bleeding phenotype	Delivery	APH/PPH	Prophylaxis
Kreuziger et al. [5]	2013	62 women	5.5% (Median)	Variable	53 vaginal 31 caesareans	PPH 10% with prophylaxis PPH 15% without prophylaxis	rFVIIa-FFP
Yazicioglu et al. [7]	2013	1	35%-46%	NA	Cesarean	APH	NA
Das and Ciantar [8]	2014	1	2	Positive	Vaginal	No	rFVIIa, tranexamic acid
Lee et al. [9]	2014	1	1.5%-11%	Positive	Vaginal	PPH in previous pregnancy	FFP
Pinar et al. [10]	2015	1	1%	Positive	Vaginal	PPH	FFP
Pfrepper et al. [11]	2017	1	1%	Positive	Vaginal	No PPH	rfVIIa
Hasoon and Rivers [12]	2019	1	21%	Positive	Vaginal	PPH	Not given
Loddo et al. [13]	2019	1	18%	Positive	Vaginal	No PPH	rfVIIa
Lee et al. [14]	2020	5 (6 pregnancies)	Median 10%	Positive	Vaginal (3) and cesarean (3)	2 PPH (1 vaginal delivery and 1 cesarean section)	rfVIIa in 2 patients and FFP in 1
Hajjar et al. [15]	2021	1 (2 pregnancies)	40%	Positive	1 abortion and 1 cesarean	Postabortal hemorrhage	rfVIIa
Rohilla et al. [16]	2021	2	<1% and 17%	positive	1 cesarean and 1 vaginal	No PPH	rfVIIa and FFPs
Yang et al. [17]	2021	1	4%	Negative	Vaginal	No PPH	rfVIIa
Obore et al. [18]	2023	1	3%	Negative	Cesarean	No PPH	rfVIIa

In the bleeding diathesis group, obstetric hemorrhage was observed in 1/3 (33.33%) patients. Although the bleeding group had a lower mean FVII level (0.75%), and pre-eclampsia could act as a confounding factor in the one case with toxemic abruption, the history of bleeding is emerging as a stronger predictor of hemorrhage risk in patients with severe FVII deficiency than FVII levels alone. Some authors have urged the use of prophylaxis in bleeding subsets of such patients in the past [6-7]. The meta-analysis by Kreuziger et al. reported equivalent hemorrhage rates between women who did and did not receive prophylaxis (Table 2) [5]. Tissue factor, von Willebrand factor, platelets, and other unknown regulators can contribute to the bleeding tendency in FVII deficiency. There may be other confounding factors in pregnancy like atonic PPH, gestational thrombocytopenia, liver disease-associated coagulopathy, and disseminated intravascular coagulation attributable to sepsis, but none of these were found in our patients.

Another concern in the management of these patients is the use of general over regional anesthesia. There is a predilection for giving general anesthesia in patients who have low FVII levels in the literature. All our patients had severe FVII deficiency (FVII levels < 10%), and all the cesarean sections were conducted under general anesthesia. Postoperative pharmacological thromboprophylaxis was avoided. Coagulation screening of babies was advised. Some babies may have bleeding from the umbilical stump, although no such episode was observed in our study. In cases where there is a history of severe, life-threatening bleeding episodes in the family, perinatal diagnosis may involve performing cordocentesis at 17-21 weeks of gestation.

Due to the rarity of these coagulation disorders, fewer than 100 cases have been reported worldwide. A case series of nine pregnancies from our population represents the largest reported. The limitation of this study is the lack of sufficient evidence to make definitive recommendations, emphasizing the importance of case-based, individualized management. A multidisciplinary team, including an obstetrician, hematologist, anesthesiologist, and neonatologist, collaborated to create an appropriate treatment plan for our patients.

## Conclusions

Thus, it is reasonable to screen all pregnant women with a coagulation profile and confirm FVII levels for those found to be deficient. However, prophylaxis should not be administered indiscriminately to all patients with severe deficiency. Blood and blood products (FFPs) and rfVIIa should be arranged in advance during the peripartum period, but transfusion instead should be individualized on a case-to-case basis. Factors like the history of bleeding, FVII levels, mode of delivery, and other risk factors for hemorrhage should all be considered before deciding on the administration of prophylaxis. Cesarean section is a surgical procedure, and prophylactic use of rfVIIa should be considered, especially in patients with severe deficiency. Route of anesthesia and postpartum thromboprophylaxis should be individualized, and prenatal diagnosis should be considered in patients/families with severe bleeding history.
